# Acute exacerbation prediction of COPD based on Auto-metric graph neural network with inspiratory and expiratory chest CT images

**DOI:** 10.1016/j.heliyon.2024.e28724

**Published:** 2024-03-29

**Authors:** Shicong Wang, Wei Li, Nanrong Zeng, Jiaxuan Xu, Yingjian Yang, Xingguang Deng, Ziran Chen, Wenxin Duan, Yang Liu, Yingwei Guo, Rongchang Chen, Yan Kang

**Affiliations:** a College of Health Science and Environmental Engineering, Shenzhen Technology University, Shenzhen 518118, China; b School of Applied Technology, Shenzhen University, Shenzhen 518060, China; c The First Affiliated Hospital of Guangzhou Medical University, State Key Laboratory of Respiratory Disease, National Clinical Research Center for Respiratory Disease, Guangzhou Institute of Respiratory Health, The National Center for Respiratory Medicine, Guangzhou 510120, China; d College of Medicine and Biological Information Engineering, Northeastern University, Shenyang 110169, China; e Department of Respiratory and Critical Care Medicine, First Affiliated Hospital of Southern University of Science and Technology, Second Clinical Medical College of Jinan University, Shenzhen People's Hospital, Shenzhen Institute of Respiratory Diseases, Shenzhen 518001, China; f Engineering Research Centre of Medical Imaging and Intelligent Analysis, Ministry of Education, Shenyang 110169, China

**Keywords:** AECOPD prediction, Machine learning, AMGNN, *Radiomics*, *CNN*, Lasso, Generalized linear model

## Abstract

Chronic obstructive pulmonary disease (COPD) is a widely prevalent disease with significant mortality and disability rates and has become the third leading cause of death globally. Patients with acute exacerbation of COPD (AECOPD) often substantially suffer deterioration and death. Therefore, COPD patients deserve special consideration regarding treatment in this fragile population for pre-clinical health management. Based on the above, this paper proposes an AECOPD prediction model based on the Auto-Metric Graph Neural Network (AMGNN) using inspiratory and expiratory chest low-dose CT images. This study was approved by the ethics committee in the First Affiliated Hospital of Guangzhou Medical University. Subsequently, 202 COPD patients with inspiratory and expiratory chest CT Images and their annual number of AECOPD were collected after the exclusion. First, the inspiratory and expiratory lung parenchyma images of the 202 COPD patients are extracted using a trained ResU-Net. Then, inspiratory and expiratory lung *Radiomics* and *CNN* features are extracted from the 202 inspiratory and expiratory lung parenchyma images by Pyradiomics and pre-trained Med3D (a heterogeneous 3D network), respectively. Last, *Radiomics* and *CNN* features are combined and then further selected by the Lasso algorithm and generalized linear model for determining node features and risk factors of AMGNN, and then the AECOPD prediction model is established. Compared to related models, the proposed model performs best, achieving an accuracy of 0.944, precision of 0.950, F1-score of 0.944, ad area under the curve of 0.965. Therefore, it is concluded that our model may become an effective tool for AECOPD prediction.

## 1 Introduction

Chronic obstructive pulmonary disease (COPD) is a prevalent, incurable respiratory disease with high morbidity, disability, and mortality rates globally [1,2]. In 2022, it has become the third leading cause of death worldwide [3,4]. Patients with acute exacerbation of COPD (AECOPD) are a significant determinant of mortality [[5], [6], [7]]. COPD patients with the following symptoms such as increased dyspnea, coughing, and sputum production indicate AECOPD, which requires special treatment attention [[8], [9], [10]]. Additionally, experiencing an AECOPD episode increases the likelihood of another exacerbation [11,12]. Therefore, it is crucial to accurately predict AECOPD in managing COPD patients.

Currently, AECOPD diagnosis requires the exclusion of other possible diagnoses through clinical and laboratory investigations [12]. For instance, clinical manifestations are currently relied upon by doctors for diagnosis, as there is no single biomarker for AECOPD clinical diagnosis and evaluation. The primary task of AECOPD diagnosis is to identify COPD. General practitioners use the Pulmonary Function Test (PFT), the gold standard for diagnosing COPD [13]. However, PFT has its limitations in that patient cooperation is required and PFT parameters cannot reflect the structural morphology of lung tissue [14,15]. Thus, the PFT may lose effectiveness for diagnosing COPD or evaluating AECOPD. Compared to PFT, chest computed tomography (CT) has become the most effective tool for quantitative analysis and characterization of COPD [16], offering advantages such as objectively and comprehensively identifying emphysema, pulmonary bronchopathy, and air trapping [17]. Likewise, chest CT has also become a primary imaging tool for diagnosing AECOPD. However, the lack of necessary dynamic information of single inspiratory chest CT images makes it insufficient for diagnosing AECOPD.

Multi-phase CT is an essential recent approach in disease research that integrates comparing CT images of different phases to obtain more detailed information and analyze them in multiple dimensions [18,19]. This approach has been proven useful in diagnosing, evaluating, and localizing lesions in a dynamic way [[20], [21], [22]]. Inspiratory and expiratory chest CT scans the entire lung from the apex to the base at the end of deep inspiration and expiration [23], providing dynamic chest CT images. Several studies have demonstrated that analyzing the difference between the inspiratory and expiratory phases is crucial for effectively characterizing COPD, showing additional information (such as the presence of air trapping) not present in single respiratory chest CT images [[24], [25], [26], [27]]. This is a hallmark of COPD that compensates for the dynamic information of single inspiratory chest CT images. Traditional statistical methods in previous studies of inspiratory and expiratory chest CT images limit further research on COPD. For example, in 2017, the parametric response mapping (PRM) method based on inspiratory and expiratory chest CT images was proposed [28], which can potentially improve COPD diagnosis by highlighting small airway lesions, emphysema, and healthy lung regions. However, the PRM method has several limitations, such as the requirement to register expiratory and inspiratory chest CT images and the location of small airway lesions, emphysema, and healthy regions according to particular threshold intervals. However, setting the threshold intervals is time-consuming and highly influenced by experience, without guidelines provided [29].

*Radiomics* technology has become one of the most commonly used feature extraction methods of medical images, freeing from experience influence. Since its introduction, radiomics has been extensively used in CT images with hundreds of millions of pixels to extract features [30]. Specifically, in COPD research, radiomics has shown promising potential in diagnosis and treatment, with its recognized usefulness [31,32]. A recent study shows that radiomics achieves near-to-standard-dose CT in predicting COPD disease on low-dose CT [33]. However, *Radiomics* technology requires annotating the region of Interest (ROI) in chest CT images, which is a challenging task in CT images with so many slices. Artificial intelligence (AI) technology has effectively solved this challenge of ROI in chest CT images in *Radiomics*. However, because radiomics features are limited by specific calculation equations, convolutional neural network (CNN) features should be considered to compensate for the limitations of radiomics features [34]. Med3D [35], a pre-trained 3D convolutional network, is a backbone for *CNN* feature extraction, which is effective for feature extraction [[36], [37], [38]].

The performance of deep learning (DL) models is limited by the volume, uneven quality, and higher inter-class similarity of medical images. Therefore, this makes it challenging for deep learning models [39,40]. Meanwhile, the performance of Graph Neural Networks (GNN) [41] has surpassed the classical DL models, such as CNN and Long-Short Term Memory networks in terms of medical [42]. However, GNN is computationally expensive and challenging to implement in real-time applications. To address the limitations of GNN, an Auto-Metric Graph Neural Network (AMGNN), a novel approach that combines GNN with meta-learning strategies, was proposed [43]. Specifically, AMGNN creates flexible graphs with more minor memory requirements, inhibits over-fitting and under-fitting phenomena, and is less sensitive to uneven data distribution [44,45].

Having the above-described motivation, this paper proposes an AECOPD prediction model based on AMGNN using the *Radiomics* and *CNN* features of inspiratory and expiratory chest low-dose CT. Our experiments showed significant improvements in AMGNN's performance compared to traditional GNN and DL methods. Our presented work makes several significant contributions.

1) Demonstrating a high accuracy rate of 94% in predicting AECOPD outcome. By applying the proposed model in clinical settings, physicians can be better equipped to prepare for AECOPD occurrences in advance, modify existing treatment plans, and potentially suppress disease exacerbations, which can help reduce economic burdens and improve patient outcomes.
2) The proposed method eliminates the need for registration using inspiratory and expiratory chest CT images. It achieves high accuracy rates and is fully automated without the need for human parameter settings, providing a highly reproducible and efficient approach. These results suggest promising new avenues for future research utilizing inspiratory and expiratory chest CT images.
3) Our experiments demonstrate the feasibility of the meta-learning strategy in the medical field. The advantages of this approach are that it can effectively target medical imaging data and outperform traditional machine learning (ML) models in COPD research, potentially making a significant contribution to the analysis of medical data.

## 2 Materials and methods

### 2.1 Materials

This study is approved by the Ethics Committee in the First Affiliated Hospital of 10.13039/100009659Guangzhou Medical University (Grant number:2017-22) and registered at the National Center for Biotechnology Information (https://www.clinicaltrials.gov/study/NCT03240315, registration number: NCT03240315). All patients participating in the study were provided with written informed consent before the PFT, chest CT scans, and questionnaires.

**Fig. 1:**
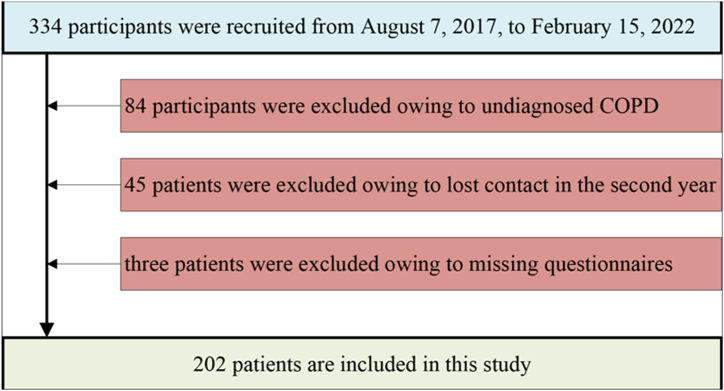
The selection process of the study cohort.

**Fig. 2:**
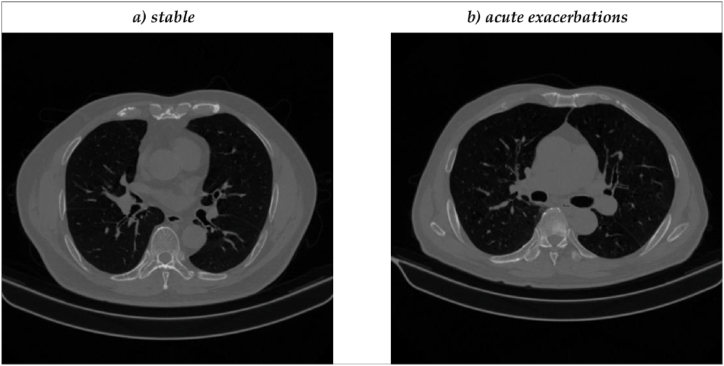
The inspiratory and expiratory low-dose CT images of two patients who stabilized in the second year and experienced an acute exacerbation. a) The CT image of a stable COPD patient. b) The CT image of an AECOPD patient.

Fig. 1 shows the selection process of the study cohort. Specifically, 334 participants were recruited from August 7, 2017, to February 15, 2022. All 334 participants performed the PFT and underwent chest CT scans for the first year. Then, these 334 participants took questionnaires every subsequent year to determine the number of AECOPD incidents. However, 84 participants were excluded owing to undiagnosed COPD by PFT. Meanwhile, 45 patients who lost contact in the second year and three patients with missing questionnaires were also excluded from this study. Finally, 202 patients are included in this study. Table 1 shows information about the 202 patients and the corresponding CT images. The inspiratory and expiratory low-dose CT images and the number of AECOPD incidents of these 202 patients were collected. 70 patients of these 202 patients experienced an increase in the number of AE in the second year by questionnaires, while 132 patients remained stable without the increase. The size of inspiratory and expiratory low-dose CT images of these 202 patients was 512 × 512 from a maximum of 409 slices to a minimum of 116 slices (an average of 343 slices). Fig. 2(a and b) shows the inspiratory and expiratory low-dose CT images of two patients who stabilized(Fig. 2 a)) in the second year and experienced an acute exacerbation(Fig. 2 b)). However, it is hard to notice the significant differences between the two patients from a visual perspective.

**Table 1:** Description of our dataset used in the study.

Label	Stable		Exacerbation	
Phase	Inspiration	Expiration	Inspiration	Expiration
Number	132		70	
Age,mean ± SD	65.26 ± 8.13		65.66 ± 7.66	
Sex(%),male(n)	92.4(122)		92.9(65)	
BMI, mean ± SD	22.24 ± 4.06		22.37 ± 2.91	
KVp(kv)	110	110	110	110
Slice thickness(mm)	1	1	1	1
X-ray tube current(mA),mean ± SD	65.82 **±** 7.75	66.04 ± 7.89	65.99 ± 6.76	66.1 ± 6.89
Exposure(mA*s),mean ± SD	30.42 **±** 3.44	30.69 ± 4.53	30.6 ± 3.59	30.57 ± 3.46

### 2.2 Methods

**Fig. 3:**
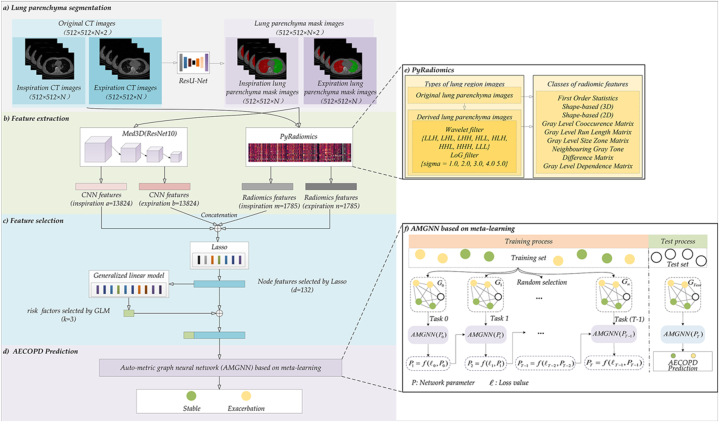
The specific flowchart for AECOPD prediction based on AMGNN with inspiratory and expiratory chest low-dose CT images. a) The lung parenchyma segmentation. b) The *CNN* and *Radiomics* feature extraction. c) Feature selection. d) AECOPD prediction based on AMGNN. f) AMGNN's meta-learning training strategy.

Fig. 3 shows the specific flowchart for AECOPD prediction based on AMGNN with inspiratory and expiratory chest low-dose CT images, including lung parenchyma segmentation, feature extraction, feature selection, and AMGNN.

#### 2.2.1 Lung parenchyma segmentation

**Fig. 4:**
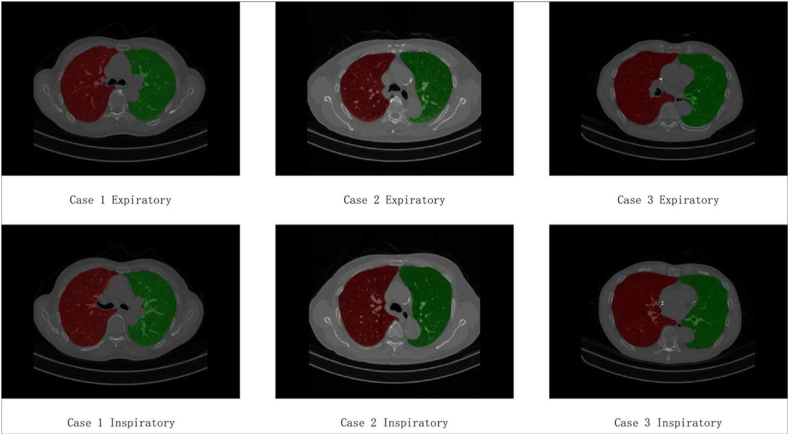
The typical lung parenchyma mask images of inspiratory and expiratory chest low-dose CT images.

Fig. 3 a) depicts a validated and pre-trained segmentation model, ResU-Net, for lung parenchyma segmentation from inspiratory and expiratory chest low-dose CT images [46]. Specifically, the inspiratory and expiratory chest low-dose CT images of each patient are separately inputted into the pre-trained ResU-Net to generate lung parenchyma mask images. Fig. 4 demonstrates the typical lung parenchyma mask images of inspiratory and expiratory chest low-dose CT images. Subsequently, the lung parenchyma mask images and their original CT images are used to extract *Radiomics* and *CNN* features.

#### 2.2.2 Feature extraction

Fig. 3 b) depicts the feature extraction process of lung parenchyma features, including *Radiomics* and *CNN* features. Specifically, PyRadiomics [30] extracts *Radiomics* features based on lung parenchyma, and a pre-trained Med3D network extracts *CNN* features based on lung parenchyma images (512 × 512 × N).

To this end, the pre-trained Med3D network is invoked to act as the backbone [35]. The Med3D network is a 3D *CNN* architecture trained on large and multiple medical images to extract robust features. The inspiratory and expiratory lung parenchyma CT images are directly fed into the Med3D to extract *CNN* features, yielding 13824 features for each phase (each patient's inspiratory phase and expiratory phase).

Fig. 3 b) and e) show the *Radiomics* feature extraction process by PyRadiomics, including two steps. Specifically, the first step involves the application of wavelet and Gaussian Laplacian filters to the original lung parenchymal image, generating derived lung parenchymal images. Then, the derived lung parenchymal images and their mask images are used to calculate *Radiomics* features based on pre-defined equations, generating 1785 features for each phase. For a more comprehensive understanding of the PyRadiomics, refer to our previous research [36,47].

#### 2.2.3 Feature selection

##### 2.2.3.1 Lasso algorithm for feature selection

Numerous studies have been conducted to determine the effectiveness of using selected features to create models. However, the results consistently show that using selected features produces better outcomes [47,48]. Our previous research [34] found that the Lasso algorithm is the most effective for feature selection. Fig. 3 c) shows each patient's *CNN* and *Radiomics* features of the inspiration and expiratory phases are concatenated separately, and then the concatenated features are subjected to feature selection using the Lasso algorithm with ten-fold cross-validation [49].

The Lasso algorithm stands as an effective feature selection method in the medical field and has been widely validated in various applications. In this study, out of a total of each patient's 31,218 (13,824 + 13,824 + 1785 + 1785) features, 132 features were automatically selected by the Lasso algorithm. Mathematically, the Lasso algorithm can be expressed by Equation (1). This algorithm identifies a subset of features most relevant to the AECOPD prediction task by minimizing the objective function [49].

(1) $$\overset{⌢}{\beta }\leftarrow \underset{\beta }{\mathrm{argmin}}\left\{\sum_{i=1}^{n}{\left(y_{i}-b-\sum_{j=1}^{p}\beta_{j}x_{ij}\right)}^{2}+\lambda \sum_{j=0}^{p}\left|\beta_{j}\right|\right\}$$

Where β denotes the 202 × 132 features are selected from 202 × 31218 features $x_{ij}$, 202 × 31218 denotes 202 patients with 2 × 13,824 CNN and 2 × 1785 *Radiomics* features of inspiratory and expiratory low-dose CT images, $y_{i}$ denotes the 202 × 1 dependent variable (202 patients' labels), *b* denotes the intercept, λ denotes the Lagrangian coefficient, and $\beta_{j}$ denotes the regression coefficient.

##### 2.2.3.2 GLM for risk factors selection

Fig. 3 c) shows that all the 132 selected features are utilized as inputs of the predictor after Lasso feature selection. However, the AMGNN predictor needs risk factors for the prediction task. The relevant risk factors selection is an essential step for the AMGNN predictor. Based on our previous research [34], three risk factors generated from the Generalized Linear Model (GLM) [50] resulted in the best performance. The GLM has been a widely used statistical method for modeling the relationship between the response variable and the explanatory variables. Equation (2) is the principle of GLM [51].

(2) $$L\left(\upsilon_{y_{i}}\right)=b+\sum_{j=1}^{n}\beta_{j}\cdot x_{ij}$$

Where $L\left(\upsilon_{y_{i}}\right)$ is the linear function that can relate mean $\upsilon_{y_{i}}=F\left(y_{i}\right)$ to linear predicted values $\mu =\sum_{j=1}^{n}\beta_{j}$. $y_{i}$ denotes the dependent variable (202 patients' labels), *b* represents the intercept, $\beta_{j}$ denotes the regression coefficient, and $x_{ij}$ denotes the response variable (202 × 31218 features).

#### 2.2.4 AMGNN

##### 2.2.4.1 Network architecture of AMGNN

**Fig. 5:**
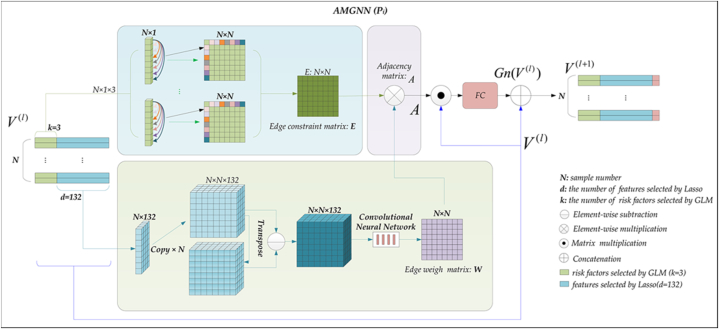
The structure of AMGNN network.

Fig. 5 details the architecture of the AMGNN predictor. The input of the AMGNN predictor is a node matrix V, which consists of risk factors and Lasso-selected features [34]. Three adjacency matrices were constructed to capture the interactions among the risk factors by computing the differences between each pair of patients within the same risk factor. These matrices are then combined into an edge constraint matrix E (N × 132) by a weighted sum. The Lasso-selected features are replicated N times to form an N × N × 132 three-dimensional matrix.

Initially, the *x* and *y* dimensions of the replicated features are subjected to a transpose operation, while the *z* dimension remains unchanged. This operation results in a matrix with the same dimensions as the adjacency matrices. Then, an element-wise subtraction is executed between the newly obtained matrix and the replicated features matrix, yielding an N × N × 132 matrix. The N × N × 132 matrix is fed into an embedded CNN to generate an edge weight matrix W. Subsequently, the edge weight matrix W is multiplied element-wise with the edge constraint matrix E, resulting in an adjacency matrix A. The node matrix V is multiplied with the adjacency matrix A, and the outcome is passed through a fully connected layer to obtain W. This process is repeated twice in the AMGNN predictor. The final output of the AMGNN predictor is obtained by inputting the result of the second fully connected layer directly into the softmax [43]. During the training process, we used the cross-entropy loss function and Adam optimizer.

#### 2.2.5 Training strategy based on meta-learning

Fig. 3 f) shows that the training strategy is based on meta-learning and involves training the predictor on small graphs with N = 21 nodes [43]. Each small graph comprises ten randomly selected stable and exacerbation cases and one unknown case. The AMGNN predictor is trained on each small graph, and after each training iteration, the predictor's parameters P are used as the initial parameters for the following small graph [43]. Using this approach, the AMGNN predictor can learn effectively from a small graph and apply the acquired knowledge to the following graphs, enhancing its performance. After training, the AMGNN predictor's initial parameters are well-trained and utilized for testing. In testing, the AMGNN predictor receives the Lasso-selected features from the test set as an unknown case in each small graph for AECOPD perdition.

## 3 Experiments and results

### 3.1 Experiments

**Fig. 6:**
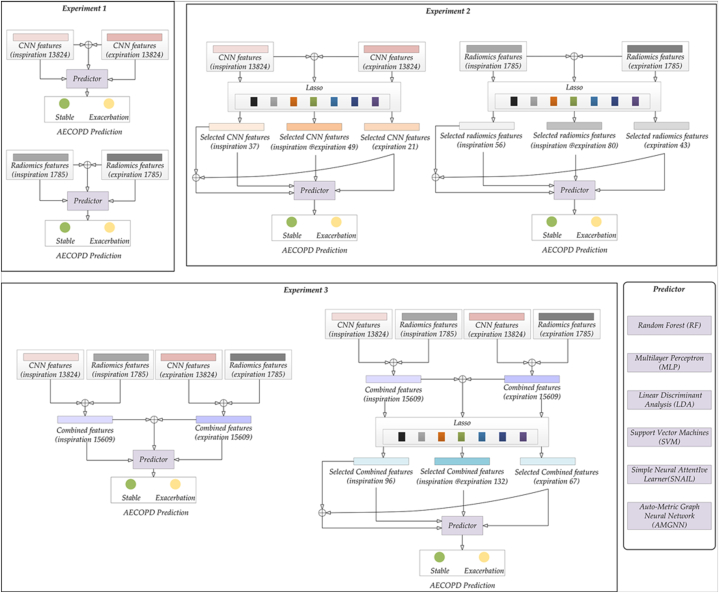
Experimental design.

Our research aims to evaluate the effectiveness of various models for predicting AECOPD and identify the best predictor. Fig. 6 illustrates that we conducted three experiments. For each experiment, we used two kinds of predictors: the traditional ML predictors and meta-learning predictors. Specifically, the traditional ML predictors include Random Forest (RF) [52], Multilayer Perceptron (MLP) [53], Linear Discriminant Analysis (10.13039/100003090LDA) [54], and Support Vector Machines (SVM) [55]. The meta-learning predictors include Simple Neural AttentIve Learner (SNAIL) [56] and AMGNN. Notably, the AMGNN predictor is augmented with risk factors selected by GLM, while the other predictors utilize the features directly.

In Experiment 1, we separately utilized expiratory (*EX*) and inspiratory (*IN*) *Radiomics*/*CNN* features without implementing Lasso feature selection. We separately fed the *EX*/*IN Radiomics*/*CNN* features and their combination (*Radiomics*/*CNN* of *EX* and *IN*) features into the two kinds of predictors. This enabled us to compare the inspiratory and expiratory chest CT and single-phase CT results. Experiment 1 served as served as an initial control group for the subsequent Experiments 2–3. In Experiment 2, we separately utilized *EX*/*IN Radiomics*/*CNN* features and *Radiomics*/*CNN* features of *EX* and *IN* after applying the Lasso algorithm (Lasso feature-selected *Radiomics*/*CNN* features) to feed into these predictors for comparing the selected *EX*/*IN Radiomics*/*CNN* features. Additionally, we applied the lasso algorithm to the combined features from the *EX* and *IN* phases in different ways to determine the best combination approach. Experiment 3 combined *EX* and *IN Radiomics* and *CNN* features to evaluate their complementarity. We compared the Lasso feature-selected and unselected *Radiomics* + *CNN* features with Experiments 1 and 2. We also compared the *EX* and *IN*, as well as their combination features, to explore the optimal use of chest CT features. In each part of Experiments 1–3, we used 5-fold cross-validation to assess the predictor's performance. For convenience, we have replaced specific feature names with the following words in Table 2. These features are collectively referred to as "phase features".

**Table 2:** Abbreviation Description.

Phase features	Specific meaning
IN	Inspiration
EX	Expiration
EX + IN	The combination of expiration and inspiration features
EX_lasso	Features obtained by using lasso to select expiration features
IN_lasso	Features obtained by using lasso to select inspiration features
EX_lasso + IN_lasso	combination of expiration features selected by lasso and inspiration features selected by lasso
(EX + IN)_lasso	The features obtained by combining expiration and inspiration features and selected through lasso

### 3.2 Results

We employed four standard metrics to thoroughly evaluate the experimental outcomes, including the accuracy, precision, F1-score, and Area Under Curve (AUC). These evaluation metrics were determined via 5-fold cross-validation and depicted their mean values. Specifically, we randomly and uniformly divided the data into five equal parts based on the labels before the experiment. There were five separate CV iterations, each iteration of the CV involved using one part as the test set and the remaining four parts as the training set. The five parts of the data were rotated as a test set, all models are re-initialized and re-trained in each CV iteration. The results from these five CV iterations were aggregated to ensure robustness in our evaluations. The results of our experiments are presented in Table 3, Table 4, Table 5. Notably, the most exceptional AECOPD prediction performance in the AMGNN was accomplished by combining the EX and IN *CNN + Radiomics* features selected by Lasso, achieving an accuracy of 0.944, precision of 0.950, F1-score of 0.944, and AUC of 0.965, respectively.

**Table 3:** Mean evaluation metrics with a standard deviation of the six AECOPD prediction models in Experiment 1.

Predictor	Phase	Accuracy	Precision	F1-score	AUC
RF^1^	EX^a^	0.589(±0.034)/0.639(**±**0.044)	0.551(**±**0.038)/0.597(**±**0.074)	0.556(**±**0.028)/0.592(**±**0.051)	0.584(**±**0.051)/0.636(**±**0.052)
	IN^b^	0.589(**±**0.037)/0.639(**±**0.059)	0.551(**±**0.038)/0.597(**±**0.086)	0.556(**±**0.033)/0.592(**±**0.052)	0.584(**±**0.046)/0.636(**±**0.040)
	EX + IN^c^	0.654(**±**0.025)/0.545(**±**0.033)	0.556(**±**0.112)/0.503(**±**0.028)	0.559(**±**0.049)/0.516(**±**0.025)	0.651(**±**0.067)/0.557(**±**0.059)
MLP^2^	EX^a^	0.569(**±**0.040)/0.584(**±**0.051)	0.556(**±**0.042)/0.567(**±**0.052)	0.561(**±**0.040)/0.573(**±**0.050)	0.568(**±**0.049)/0.588(**±**0.065)
	IN^b^	0.555(**±**0.029)/0.600(**±**0.082)	0.536(**±**0.022)/0.580(**±**0.089)	0.543(**±**0.024)/0.585(**±**0.082)	0.552(**±**0.058)/0.626(**±**0.094)
	EX + IN^c^	0.663(**±**0.033)/0.584(**±**0.030)	0.607(**±**0.117)/0.582(**±**0.026)	0.584(**±**0.042)/0.582(**±**0.027)	0.681(**±**0.027)/0.618(**±**0.041)
LDA^3^	EX^a^	0.585(**±**0.034)/0.570(**±**0.046)	0.583(**±**0.027)/0.577(**±**0.061)	0.582(**±**0.029)/0.571(**±**0.048)	0.609(**±**0.031)/0.604(**±**0.033)
	IN^b^	0.564(**±**0.055)/0.639(**±**0.049)	0.555(**±**0.055)/0.636(**±**0.041)	0.559(**±**0.055)/0.636(**±**0.045)	0.526(**±**0.061)/0.653(**±**0.064)
	EX + IN^c^	0.663(**±**0.011)/0.554(**±**0.018)	0.585(**±**0.137)/0.566(**±**0.024)	0.552(**±**0.029)/0.559(**±**0.020)	0.688(**±**0.027)/0.563(**±**0.043)
SVM^4^	EX^a^	0.619(**±**0.038)/0,614(**±**0.035)	0.446(**±**0.028)/0.528(**±**0.078)	0.511(**±**0.003)/0.548(**±**0.046)	0.650(**±**0.032)/0.648(**±**0.022)
	IN^b^	0.644(**±**0.010)/0.668(**±**0.053)	0.449(**±**0.045)/0.603(**±**0.172)	0.519(**±**0.010)/0.579(**±**0.071)	0.644(**±**0.033)/0.700(**±**0.042)
	EX + IN^c^	0.653(**±**0.007)/0.653(**±**0.031)	0.427(**±**0.009)/0.581(**±**0.146)	0.516(**±**0.008)/0.547(**±**0.044)	0.701(**±**0.041)/0.671(**±**0.039)
SNAIL^5^	EX^a^	0.699(**±**0.044)/0.689(**±**0.028)	0.720(**±**0.039)/0.687(**±**0.024)	0.700(**±**0.041)/0.672(**±**0.021)	0.669(**±**0.067)/0.656(**±**0.044)
	IN^b^	0.648(**±**0.026)/0.718(**±**0.027)	0.679(**±**0.038)/0.715(**±**0.037)	0.655(**±**0.027)/0.710(**±**0.039)	0.605(**±**0.038)/0.708(**±**0.027)
	EX + IN^c^	0.654(**±**0.054)/0.713(**±**0.042)	0.720(**±**0.087)/0.713(**±**0.049)	0.656(**±**0.056)/0.712(**±**0.045)	0.640(**±**0.077)/0.692(**±**0.081)
AMGNN^6^	**EX**^a^	**0.659(±0.022)/0.718(±0.021)**	**0.699(±0.032)/0.729(±0.031)**	**0.663(±0.022)/0.710(±0.030)**	**0.646(±0.030)/0.686(±0.026)**
	**IN**^b^	**0.648(±0.062)/0.738(±0.037)**	**0.676(±0.073)/0.738(±0.037)**	**0.654(±0.062)/0.732(±0.040)**	**0.609(±0.047)/0.749(±0.080)**
	**EX + IN**^c^	**0.778(±0.035)/0.700(±0.083)**	**0.822(±0.013)/0.693(±0.085)**	**0.718(±0.066)/0.694(±0.082)**	**0.780(±0.040)/0.639(±0.090)**

P_6,1_ = 1.63 × 10^−28^, P_6,2_ = 5.46 × 10^−30^, P_6,3_ = 1.74 × 10^−26^, P_6,4_ = 1.81 × 10^−16^, P_6,5_ = 1.8 × 10^−4^, P_c,a_ = 4.08 × 10^−12^, P_c,b_ = 9.91 × 10^−20^, as derived from a T-test.

**Table 4:** Mean evaluation metrics with a standard deviation of the six AECOPD prediction models in Experiment 2.

Predictor	Phase	Accuracy	Precision	F1-score	AUC
RF^1^	EX_lasso^d^	0.663(**±**0.055)/0.604(**±**0.049)	0.634(**±**0.074)/0.555(**±**0.075)	0.630(**±**0.068)/0.551(**±**0.041)	0.700(**±**0.033)/0.640(**±**0.043)
	IN_lasso^e^	0.717(**±**0.071)/0.604(**±**0.033)	0.708(**±**0.084)/0.557(**±**0.037)	0.695(**±**0.076)/0.559(**±**0.024)	0.787(**±**0.075)/0.637(**±**0.040)
	EX_lasso + IN_lasso^f^	0.749(**±**0.069)/0.634(**±**0.051)	0.749(**±**0.090)/0.614(**±**0.086)	0.727(**±**0.077)/0.583(**±**0.054)	0.795(**±**0.091)/0.675(**±**0.035)
	(EX + IN)_lasso^g^	0.762(**±**0.035)/0.614(**±**0.041)	0.770(**±**0.042)/0.567(**±**0.103)	0.736(**±**0.044)/0.565(**±**0.044)	0.823(**±**0.062)/0.668(**±**0.061)
MLP^2^	EX_lasso^d^	0.723(**±**0.038)/0.668(**±**0.048)	0.716(**±**0.040)/0.659(**±**0.051)	0.716(**±**0.038)/0.662(**±**0.050)	0.761(**±**0.023)/0.727(**±**0.065)
	IN_lasso^e^	0.742(**±**0.027)/0.717(**±**0.063)	0.745(**±**0.029)/0.712(**±**0.067)	0.741(**±**0.027)/0.717(**±**0.063)	0.833(**±**0.058)/0.770(**±**0.045)
	EX_lasso + IN_lasso^f^	0.808(**±**0.079)/0.782(**±**0.053)	0.806(**±**0.082)/0.780(**±**0.059)	0.806(**±**0.081)/0.776(**±**0.049)	0.895(**±**0.071)/0.805(**±**0.042)
	(EX + IN)_lasso^g^	0.871(**±**0.042)/0.719(**±**0.010)	0.871(**±**0.044)/0.717(**±**0.011)	0.869(**±**0.044)/0.710(**±**0.013)	0.934(**±**0.032)/0.787(**±**0.048)
LDA^3^	EX_lasso^d^	0.733(**±**0.015)/0.647(**±**0.089)	0.725(**±**0.019)/0.634(**±**0.102)	0.721(**±**0.023)/0.628(**±**0.085)	0.816(**±**0.020)/0.716(**±**0.063)
	IN_lasso^e^	0.811(**±**0.062)/0.742(**±**0.040)	0.809(**±**0.065)/0.737(**±**0.040)	0.809(**±**0.063)/0.738(**±**0.039)	0.871(**±**0.060)/0.800(**±**0.042)
	EX_lasso + IN_lasso^f^	0.803(**±**0.054)/0.787(**±**0.032)	0.800(**±**0.056)/0.792(**±**0.041)	0.800(**±**0.054)/0.784(**±**0.031)	0.882(**±**0.052)/0.843(**±**0.014)
	(EX + IN)_lasso^g^	0.861(**±**0.054)/0.748(**±**0.036)	0.863(**±**0.058)/0.746(**±**0.030)	0.860(**±**0.054)/0.740(**±**0.035)	0.934(**±**0.032)/0.813(**±**0.057)
SVM^4^	EX_lasso^d^	0.778(**±**0.045)/0.697(**±**0.047)	0.776(**±**0.045)/0.696(**±**0.078)	0.772(**±**0.045)/0.648(**±**0.052)	0.815(**±**0.043)/0.766(**±**0.038)
	IN_lasso^e^	0.817(**±**0.025)/0.752(**±**0.042)	0.823(**±**0.029)/0.759(**±**0.046)	0.810(**±**0.024)/0.724(**±**0.050)	0.887(**±**0.035)/0.805(**±**0.047)
	EX_lasso + IN_lasso^f^	0.813(**±**0.090)/0.732(**±**0.038)	0.811(**±**0.097)/0.752(**±**0.051)	0.808(**±**0.095)/0.699(**±**0.058)	0.903(**±**0.068)/0.822(**±**0.021)
	(EX + IN)_lasso^g^	0.851(**±**0.033)/0.728(**±**0.033)	0.853(**±**0.037)/0.731(**±**0.042)	0.847(**±**0.033)/0.687(**±**0.049)	0.939(**±**0.013)/0.819(**±**0.050)
SNAIL^5^	EX_lasso^d^	0.832(**±**0.033)/0.783(**±**0.069)	0.843(**±**0.037)/0.786(**±**0.074)	0.831(**±**0.032)/0.779(**±**0.070)	0.845(**±**0.040)/0.768(**±**0.073)
	**IN_lasso**^e^	**0.875(±0.046)/0.806(±0.055)**	**0.878(±0.047)/0.805(±0.057)**	**0.875(±0.046)/0.802(±0.055)**	**0.901(±0.039)/0.794(±0.071)**
	**EX_lasso + IN_lasso**^f^	**0.897(±0.056)/0.823(±0.046)**	**0.897(±0.057)/0.824(±0.048)**	**0.896(±0.057)/0.820(±0.047)**	**0.924(±0.054)/0.803(±0.064)**
	**(EX + IN)_lasso**^g^	**0.926(±0.027)/0.842(±0.029)**	**0.929(±0.027)/0.843(±0.028)**	**0.926(±0.027)/0.842(±0.029)**	**0.939(±0.027)/0.876(±0.027)**
AMGNN^6^	**EX_lasso**^d^	**0.832(±0.033)/0.787(±0.064)**	**0.837(±0.039)/0.787(±0.063)**	**0.821(±0.031)/0.786(±0.064)**	**0.805(±0.050)/0.804(±0.082)**
	IN_lasso^e^	0.871(**±**0.041)/0.811(**±**0.053)	0.878(**±**0.043)/0.814(**±**0.051)	0.868(**±**0.045)/0.810(**±**0.051)	0.877(**±**0.037)/0.814(**±**0.054)
	EX_lasso + IN_lasso^f^	0.872(**±**0.069)/0.818(**±**0.047)	0.873(**±**0.073)/0.826(**±**0.053)	0.870(**±**0.070)/0.817(**±**0.047)	0.896(**±**0.064)/0.821(**±**0.054)

Predictor	Phase	Accuracy	Precision	F1-score	AUC
AMGNN	(EX + IN)_lasso^g^	0.921(**±**0.024)/0.822(**±**0.025)	0.922(**±**0.024)/0.821(**±**0.026)	0.921(**±**0.024)/0.820(**±**0.026)	0.938(**±**0.011)/0.832(**±**0.025)

P_6,1_ = 2.13 × 10^−70^, P_6,2_ = 3.35 × 10^−26^, P_6,3_ = 7.92 × 10^−21^, P_6,4_ = 1.12 × 10^−15^, P_6,5_ = 0.0421, P_g,d_ = 5.89 × 10^−39^, P_g,e_ = 1.37 × 10^−13^, P_g,f_ = 1.27 × 10^−6^ as derived from a T-test.

**Table 5:** Mean evaluation metrics with a standard deviation of the six AECOPD prediction models in Experiment 3.

Predictor	Phase	Accuracy	Precision	F1-score	AUC
RF^1^	EX^a^	0.610(**±**0.052)	0.583(**±**0.114)	0.558(**±**0.043)	0.637(**±**0.037)
	IN^b^	0.618(**±**0.041)	0.577(**±**0.053)	0.582(**±**0.048)	0.637(**±**0.012)
	EX + IN^c^	0.599(**±**0.052)	0.547(**±**0.056)	0.552(**±**0.046)	0.608(**±**0.098)
	EX_lasso^d^	0.693(**±**0.030)	0.686(**±**0.043)	0.672(**±**0.027)	0.753(**±**0.064)
	IN_lasso^e^	0.722(**±**0.070)	0.730(**±**0.093)	0.696(**±**0.070)	0.791(**±**0.035)
	EX_lasso + IN_lasso^f^	0.748(**±**0.028)	0.757(**±**0.022)	0.718(**±**0.043)	0.802(**±**0.022)
	(EX + IN)_lasso^g^	0.753(**±**0.044)	0.769(**±**0.057)	0.718(**±**0.053)	0.772(**±**0.055)
MLP^2^	EX^a^	0.594(**±**0.019)	0.568(**±**0.028)	0.576(**±**0.025)	0.594(**±**0.076)
	IN^b^	0.545(**±**0.010)	0.519(**±**0.028)	0.528(**±**0.020)	0.559(**±**0.057)
	EX + IN^c^	0.584(**±**0.045)	0.564(**±**0.043)	0.571(**±**0.043)	0.596(**±**0.063)
	EX_lasso^d^	0.747(**±**0.062)	0.743(**±**0.066)	0.742(**±**0.063)	0.839(**±**0.041)
	IN_lasso^e^	0.836(**±**0.041)	0.839(**±**0.045)	0.832(**±**0.044)	0.906(**±**0.027)
	EX_lasso + IN_lasso^f^	0.856(**±**0.020)	0.858(**±**0.018)	0.852(**±**0.023)	0.919(**±**0.026)
	(EX + IN)_lasso^g^	0.862(**±**0.039)	0.861(**±**0.041)	0.860(**±**0.040)	0.903(**±**0.026)
LDA^3^	EX^a^	0.570(**±**0.049)	0.570(**±**0.037)	0.569(**±**0.043)	0.562(**±**0.043)
	IN^b^	0.608(**±**0.035)	0.605(**±**0.030)	0.605(**±**0.032)	0.594(**±**0.040)
	EX + IN^c^	0.560(**±**0.046)	0.556(**±**0.044)	0.556(**±**0.044)	0.554(**±**0.054)
	EX_lasso^d^	0.782(**±**0.055)	0.782(**±**0.053)	0.780(**±**0.054)	0.835(**±**0.035)
	IN_lasso^e^	0.742(**±**0.098)	0.745(**±**0.099)	0.741(**±**0.098)	0.840(**±**0.058)
	EX_lasso + IN_lasso^f^	0.881(**±**0.034)	0.885(**±**0.035)	0.879(**±**0.034)	0.934(**±**0.020)
	(EX + IN)_lasso^g^	0.886(**±**0.036)	0.886(**±**0.037)	0.885(**±**0.037)	0.918(**±**0.033)
SVM^4^	EX^a^	0.638(**±**0.023)	0.459(**±**0.073)	0.517(**±**0.023)	0.636(**±**0.027)
	IN^b^	0.658(**±**0.016)	0.496(**±**0.146)	0.527(**±**0.029)	0.641(**±**0.033)
	EX + IN^c^	0.633(**±**0.011)	0.471(**±**0.055)	0.521(**±**0.014)	0.642(**±**0.036)
	EX_lasso^d^	0.801(**±**0.065)	0.805(**±**0.070)	0.786(**±**0.073)	0.866(**±**0.038)
	IN_lasso^e^	0.802(**±**0.066)	0.802(**±**0.076)	0.787(**±**0.077)	0.916(**±**0.033)
	EX_lasso + IN_lasso^f^	0.851(**±**0.016)	0.863(**±**0.024)	0.843(**±**0.016)	0.921(**±**0.010)
	(EX + IN)_lasso^g^	0.866(**±**0.038)	0.871(**±**0.043)	0.862(**±**0.040)	0.921(**±**0.037)
SNAIL^5^	**EX**^a^	**0.704(±0.036)**	**0.702(±0.042)**	**0.701(±0.039)**	**0.660(±0.050)**
	IN^b^	0.747(**±**0.025)	0.789(**±**0.034)	0.725(**±**0.028)	0.742(**±**0.030)
	EX + IN^c^	0.708(**±**0.038)	0.720(**±**0.053)	0.704(**±**0.038)	0.686(**±**0.049)
	EX_lasso^d^	0.832(**±**0.035)	0.845(**±**0.043)	0.830(**±**0.040)	0.847(**±**0.033)
	IN_lasso^e^	0.896(**±**0.025)	0.903(**±**0.017)	0.895(**±**0.023)	0.927(**±**0.019)
	EX_lasso + IN_lasso^f^	0.910(**±**0.034)	0.923(**±**0.022)	0.910(**±**0.033)	0.938(**±**0.023)
	(EX + IN)_lasso^g^	0.921(**±**0.027)	0.921(**±**0.028)	0.921(**±**0.028)	0.942(**±**0.025)
AMGNN^6^	EX^a^	0.667(**±**0.082)	0.695(**±**0.065)	0.675(**±**0.076)	0.648(**±**0.086)
	**IN**^b^	**0.789(±0.052)**	**0.783(±0.073)**	**0.772(±0.060)**	**0.733(±0.067)**
	**EX + IN**^c^	**0.744(±0.048)**	**0.737(±0.060)**	**0.731(±0.057)**	**0.713(±0.069)**
	**EX_lasso**^d^	**0.856(±0.041)**	**0.853(±0.043)**	**0.853(±0.043)**	**0.866(±0.032)**
	**IN_lasso**^e^	**0.944(±0.017)**	**0.947(±0.019)**	**0.944(±0.018)**	**0.963(±0.015)**
	**EX_lasso + IN_lasso**^f^	**0.944(±0.024)**	**0.949(±0.024)**	**0.945(±0.024)**	**0.950(±0.017)**
	**(EX + IN)_lasso**^g^	**0.944(±0.039)**	**0.950(±0.034)**	**0.944(±0.039)**	**0.965(±0.032)**

P_6,1_ = 2.19 × 10^−45^, P_6,2_ = 1.16 × 10^−18^, P_6,3_ = 8.35 × 10^−18^, P_6,4_ = 3.53 × 10^−15^, P_6,5_ = 0.049, P_g,a_ = 8.40 × 10^−108^, P_g,b_ = 5.01 × 10^−78^, P_g,c_ = 5.48 × 10^−96^, P_g,d_ = 4.31 × 10^−23^, P_g,e_ = 6.76 × 10^−6^, P_g,f_ = 0.091 as derived from a T-test.

#### 3.2.1 AECOPD prediction outcome in Experiment 1

Table 3 reports the six predictors’ performance, including the accuracy, precision, F1-score, and AUC based on *EX*, *IN,* and *EX + IN* features. It is observed that, except for the AMGNN, there is no significant difference in accuracy, precision, F1-score, and AUC between *EX Radiomics* and *EX CNN* features. The AMGNN predictor based on *Radiomics* features performs better compared to *CNN* features, improving accuracy of 5.9% accuracy, precision of 3.0%, F1-score of 4.7%, and AUC of 4.0%. Other predictors did not show such improvements. However, ML models achieved based on *EX*, *IN,* and *EX + IN* features only around 0.600 accuracy.

**Fig. 7:**
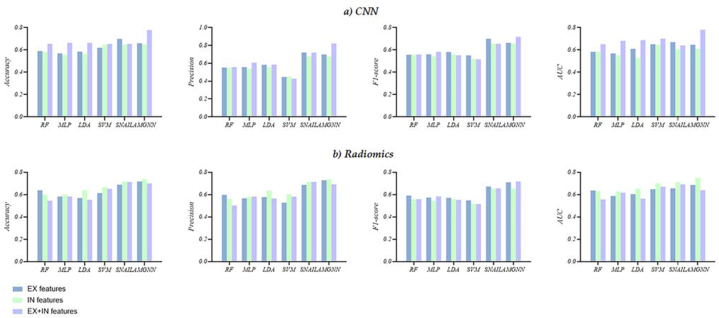
The visual evaluation metrics of the six AECOPD prediction models in Experiment 1. a) *CNN* features, b) *Radiomics* features.

All predictors performed better on *IN Radiomics* features than on *IN CNN* features. Specifically, SNAIL and AMGNN predictors outperformed the top SVM predictors on *IN Radiomics* features. However, SNAIL and AMGNN predictors based on IN CNN features did not show significant superiority over SVM predictor. There is only a slight improvement in SNAIL and AMGNN predictors' performance. The performance of predictors based on *Radiomics* and *CNN* features varies between the *EX* and *IN*. The performance of predictors based on *IN CNN* features performed better than the *EX CNN* features. Whereas, the predictors based on the *EX Radiomics* features performed slightly better than the *IN Radiomics* features. The AMGNN predictor with the *EX* + *IN CNN* features performs best, achieving an accuracy of 0.778, precision of 0.822, F1-score of 0.718, and AUC of 0.780. Additionally, the results showed that SNAIL and AMGNN outperformed traditional ML models. While the *EX* + *IN CNN* features had a better, they did not exceed the IN *Radiomics* features, as shown in Fig. 7(a and b).

#### 3.2.2 AECOPD prediction outcome in Experiment 2

In this section, we present the results of our experiments to compare Experiment 1. Table 4 reports the performance of the six predictors on *EX* and *IN* features after applying the Lasso algorithm. Our findings demonstrate that in both *EX_lasso CNN* and *EX_lasso Radiomics* features, the accuracy, precision, F1-score, and AUC of expiratory features are higher than *EX CNN* and *EX Radiomics* features.

Furthermore, *EX_lasso/IN_lasso CNN* features exhibit a more significant advantage over *EX_lasso/IN_lasso Radiomics* features, at least improving the accuracy of 2.5%, precision of 3.3%, F1-score of 2.4%, and AUC of 0.1%, respectively. The results indicate that the *IN_lasso* features exhibit *EX_lasso*. Compared to AMGNN, SNAIL based on *(EX + IN)_lasso CNN* features performs better, improving the accuracy of 0.4%, F1-score of 0.7%, and AUC of 2.4%, respectively.

**Fig. 8:**
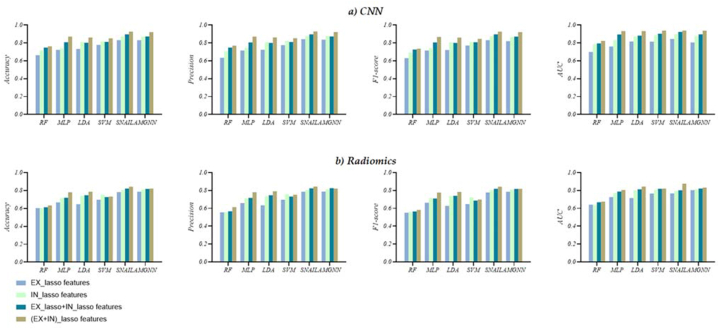
The visual evaluation metrics of the six AECOPD prediction models in Experiment 2. a) *CNN* features, b) *Radiomics* features.

The performance of both meta-learning models (SNAIL and AMGNN) is higher than the SVM model, which has the best performance among the ML models. The SNAIL predictor based on *EX_lasso + IN_lasso* features performs best, achieving an accuracy of 0.897, precision of 0.897, F1-score of 0.896, and AUC of 0.924, respectively. Compared to the SNAIL predictor based on *IN_lasso* features, the performance of the SNAIL predictor based on *EX_lasso + IN_lasso* features, improving the accuracy of 2.2%, precision of 1.9%, F1-score of 2.1%, and AUC of 2.3%, respectively. The SNAIL predictor based on *(EX + IN)_lasso* features performs best, achieving an accuracy of 0.926, precision of 0.929, F1-score of 0.926, and AUC of 0.939. Compared to the AMGNN predictor based on *(EX + IN)_lasso* features, the performance of SNAIL predictor based on *(EX + IN)_lasso* features performs better with a slight advantage of the accuracy of 0.5%, precision of 0.7%, F1-score of 0.5%, and AUC of 0.1%, respectively. Fig. 8(a and b) illustrates that regardless of *CNN* or *Radiomics* features, *EX_lasso + IN_lasso*/*(EX + IN)_lasso* features yielded better results than using any single phase (*EX, IN, EX_lasso, IN_lasso*).

#### 3.2.3 AECOPD prediction outcome in Experiment 3

Table 5 reports the experimental results of the six predictors based on *Radiomics* and *CNN* features from seven different phases are presented. The findings show that AMGNN performs better than SNAIL except for the unselected *inspiration EX* features. The AMGNN based on *(EX + IN)_lasso* features performs best, achieving the highest accuracy of 0.944, precision of 0.950, F1-score of 0.944, and AUC of 0.965, respectively.

**Fig. 9:**
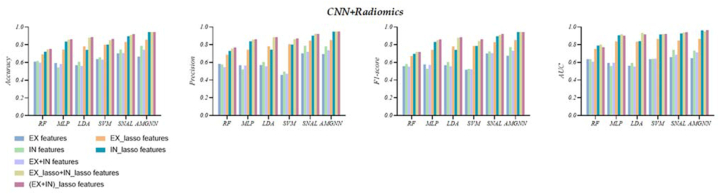
The visual evaluation metrics of the six AECOPD prediction models based on *CNN* + *Radiomics* features in Experiment 3.

Compared to the AMGNN based on *(EX + IN)_lasso* features, the SNAIL based on *(EX + IN)_lasso* features achieves an accuracy of 0.921, precision of 0.921, F1-score of 0.921, and AUC of 0.942, respectively. Meanwhile, the LDA based on *(EX + IN)_lasso* features outperformed other ML models, achieving an accuracy of 0.886, precision of 0.886, F1-score of 0.885, and AUC of 0.918, respectively. It is worth noting that all predictors based on *(EX + IN)_lasso* features achieved their best performances. In particular, the AMGNN demonstrated consistent accuracy across Experiments 1–3 after applying five-fold cross-validation. Fig. 9 provides a vertical comparison of the six predictors based on *Radiomics* and *CNN* features from seven different phases. Specifically, the *IN* features outperformed *EX* features, and this trend persisted even after applying the Lasso algorithm. *EX + IN* features did not demonstrate any significant advantages before feature selection. However, considerable improvement was observed after applying the Lasso algorithm.

#### 3.2.4 Synthesized assessment

Table 6, Table 7 report a comprehensive and quantitative comparison of different predictors, phases, and features. Specifically, Table 6 displays the average accuracy, precision, F1-score, and AUC of each predictor on different phases at the same features (*CNN/Radiomics*). The outcomes show that, under different features, all predictors' accuracy, precision, F1-score, and AUC follow the order: *Radiomics* < *CNN* < *CNN + Radiomics*. Moreover, the accuracy and AUC of the six predictors under all features follow the order: RF < MLP < LDA < SVM < SNAIL < AMGNN. On the other hand, the precision and F1-score of different predictors under all features follow the order: RF < MLP < SVM < LDA < SNAIL < AMGNN.

**Table 6:** Average of evaluation metrics obtained for all phases of each predictor on different features.

Predictor	CNN	Radiomics	CNN + Radiomics
RF^1^	0.673/0.645/0.637/0.703	0.606/0.565/0.566/0.635	0.678/0.664/0.642/0.714
MLP^2^	0.704/0.691/0.689/0.746	0.665/0.657/0.652/0.703	0.718/0.707/0.709/0.759
LDA^3^	0.717/0.703/0.698/0.761	0.670/0.670/0.653/0.713	0.718/0.718/0.716/0.748
SVM^4^	0.739/0.655/0.689/0.791	0.692/0.664/0.620/0.747	0.750/0.681/0.692/0.792
SNAIL^5^	0.790/0.809/0.791/0.789	0.768/0.768/0.747/0.757	0.817/0.829/0.812/0.820
**AMGNN**^**6**^	**0.797/0.815/0.788/0.793**	**0.770/0.773/0.759/0.764**	**0.841/0.845/0.838/0.834**

P_6,1_ = 4.30 × 10^−129^, P_6,2_ = 1.11 × 10^−49^, P_6,3_ = 8.35 × 10^−43^, P_6,4_ = 6.79 × 10^−34^, P_6,5_ = 0.091 as derived from a T-test.

**Table 7:** Average of evaluation metrics obtained for all predictors of each phase on different features.

Phase	CNN^α^	Radiomics^β^	CNN + radiomics^γ^
EX^a^	0.620/0.593/0.602/0.621	0.636/0.614/0.611/0.636	0.631/0.596/0.599/0.623
IN^b^	0.607/0.574/0.581/0.587	0.661/0.639/0.581/0.678	0.661/0.628/0.623/0.651
EX + IN^c^	0.678/0.620/0.598/0.690	0.625/0.606/0.598/0.623	0.638/0.599/0.606/0.633
EX_lasso^d^	0.760/0.755/0.749/0.790	0.698/0.686/0.676/0.737	0.785/0.786/0.777/0.834
IN_lasso^e^	0.806/0.807/0.800/0.859	0.739/0.731/0.725/0.770	0.824/0.828/0.816/0.891
EX_lasso + IN_lasso^f^	0.824/0.823/0.818/0.883	0.742/0.735/0.723/0.785	0.865/0.873/0.858/0.911
**(EX + IN)_lasso**^**g**^	**0.865/0.868/0.860/0.918**	**0.767/0.767/0.751/0.809**	**0.872/0.876/0.865/0.904**

P_g,a_ = 3.69 × 10^−41^, P_g,b_ = 1.15 × 10^−34^, P_g,c_ = 1.45 × 10^−34^, P_g,d_ = 2.05 × 10^−10^, P_g,e_ = 0.01, P_g,f_ = 0.07.

P_γ,α_ = 0.081, P_γ,β_ = 1.42 × 10^−9^ as derived from a T-test.

Specifically, Table 7 shows the mean accuracy, precision, F1-score, and AUC of all phases under the six predictors with different features. *EX, IN,* and *EX + IN* separately follow the order *CNN* < *CNN + Radiomics* < *Radiomics*. Nevertheless, *CNN* features of *EX_Lasso*, *IN_Lasso*, *EX_Lasso + IN_Lasso*, and *(EX + IN)_Lasso* significantly outperformed *Radiomics* features. The mean values of each valuation metric for all predictors under *EX_Lasso*, *IN_Lasso*, *EX_Lasso + IN_Lasso*, and *(EX + IN)_Lasso CNN* features are higher than *Radiomics* features, at least improving accuracy of 6.2%, precision of 6.9%, F1-score of 7.3%, and AUC of 5.3%, respectively. While *CNN + Radiomics* features outperform *CNN* features, at least improving an accuracy of 0.7%, precision of 0.8%, F1-score of 0.5%, and AUC of 1.4%, respectively. Conversely, there is no apparent pattern among the *EX, IN,* and *EX + IN* under *CNN, Radiomics, and CNN + Radiomics* features. However, after feature selection, they adhere to the order *EX_Lasso* < *IN_Lasso* < *EX_Lasso + IN_Lasso* < *(EX + IN)_Lasso*. These findings demonstrate that combining the two phases (*EX + IN*) with the Lasso algorithm (*EX + IN*) *_Lasso* yields the highest accuracy, while *EX* features yield the lowest accuracy. Therefore, we confirm that both the feature extraction methods based on inspiratory and expiratory chest CT images used significantly impact the predictor's performance.

Our experiment ran on the Windows 10 Pro 64-bit operating system with two 2080 Ti GPUs, 32 GB RAM, 1 TB mechanical storage, and 256G SSD. All methods were programmed in Python and implemented based on Python 3.7.7. Table 8 demonstrates the computation time of all the methods in this paper. Feature extraction methods, Med3D and PyRadiomics, demonstrated contrasting computational speeds, with Med3D exhibiting surprisingly faster performance at 15–25 s per case, outpacing PyRadiomics, which required 50–65 min per case. For the predictive modeling methods, the training times varied considerably. RF, MLP, SVM, and LDA exhibited relatively short training times of 1–3 s, 5–8 s, 3–7 s, and 1–2 s, respectively. In contrast, SNAIL and AMGNN, as advanced deep learning approaches, demanded more extensive training times. SNAIL, with a training time of 1500–1600 s, showcased commendable performance, while AMGNN achieved the best results despite a relatively shorter training time of 270–320 s.

**Table 8:** Comparison with calculation times.

Methods	Time
Med3D	15–25s/case
PyRadiomics	50–65 m/case
RF	1–3s for training
MLP	5–8s for training
LDA	1–2s for training
SVM	3–7s for training
SNAIL	1500–1700s for training
AMGNN	270–320s for training

We looked up currently published studies related to AECOPD prediction [[57], [58], [59], [60], [61], [62]] and employed these methods within the optimal group of our experiments to compare with our approaches. Additionally, we introduce the state-of-the-art GNN model NAGphormer [63], as well as the state-of-the-art 3D deep learning model for 3D medical images, 3DMSVIT [64]. Both NAGphormer and 3DMSVIT were retrained and optimized using our dataset to ensure consistency in evaluation conditions. The final results are presented in Table 9.

**Table 9:** Comparison of the results of our proposed method with previous methods.

Predictor	Accuracy	Precision	F1-score	AUC
**AMGNN**^6^	**0.944(±0.039)**	**0.950(±0.034)**	**0.944(±0.039)**	**0.965(±0.032)**
GB^7^	0.653(**±**0.007)	0.427(**±**0.009)	0.516(**±**0.008)	0.694(**±**0.024)
DT^8^	0.610(**±**0.041)	0.621(**±**0.028)	0.612(**±**0.036)	0.609(**±**0.041)
XGBoost^9^	0.658(**±**0.065)	0.640(**±**0.077)	0.639(**±**0.067)	0.686(**±**0.056)
LR^10^	0.653(**±**0.007)	0.427(**±**0.009)	0.516(**±**0.008)	0.778(**±**0.047)
NAGphormer^11^	0.625(**±**0.082)	0.596(**±**0.073)	0.601(**±**0.068)	0.653(**±**0.090)
3DMSVIT^12^	0.653(**±**0.004)	0.427(**±**0.005)	0.516(**±**0.005)	0.682(**±**0.053)

P_6,7_ = 4.72 × 10^−7^, P_6,8_ = 2.52 × 10^−6^, P_6,9_ = 6.67 × 10^−5^, P_6,10_ = 4.72 × 10^−7^, P_6,11_ = 1.08 × 10^−4^, P_6,12_ = 2.12 × 10^−5^ as derived from a T-test.

## 4 Discussion

After analyzing the experimental results, we have compiled the following discussion to summarize our empirical findings, identify any limitations in our methodology, and suggest potential directions for future research.

### 4.1 Combination of CNN and radiomics may become an effective imaging biomarker for predicting AECOPD

Our experimental findings demonstrate that the combination of *CNN* and *Radiomics* features represents the most effective approach. Overall, Med3D-extracted *CNN* features emerge as a superior choice when compared to the Pyradiomics-extracted *Radiomics* features. This is because Med3D is trained by multimodal high-volume medical images, incorporating a substantial amount of additional priori knowledge compared to unsupervised Pyradiomics [30,35]. Therefore, Med3D outperforms Pyradiomics for AECOPD prediction. However, *CNN* needs to be supplemented by *Radiomics* to characterize AECOPD comprehensively. Specifically, the results of Experiment 3 confirmed that the combination of *CNN* and *Radiomics* features is more effective than *CNN* features. This observation implies that certain information that relates to AECOPD present in the *Radiomics* features is not captured by Med3D. This is because *Radiomics* features can effectively represent the shape and texture of the lung field related to AECOPD [65]. Consequently, the combination of CNN and Radiomics may become an effective imaging biomarker for predicting AECOPD.

### *4.2* *Synergizing Inspiratory and expiratory Features for enhancing AECOPD prediction*

The combination of *IN* and *EX* features after the Lasso algorithm resulted in the highest performance for AECOPD Prediction. Overall, the *IN* features performing better than the *EX* features. Meanwhile, a combination of *IN* and *EX* features led to a better improvement compared to *IN* features. When the patient holds their breath at the end of inhalation, the entire lung is in an expanded state [66]. In the expanded state, more details of the lungs are presented, which is the reason why the *IN* features are superior to the *EX* features. Conversely, When the patient holds their breath at the end of exhalation, the entire lung is in a contracted state [66]. Expiratory chest CT images exhibit greater sensitivity in depicting the air-trapping sign [67]. This sign stands as one of the foremost indicators of lung function impairment and airflow limitation, serving as the sole valid imaging marker for discerning the extent of small airway injury and obstruction [68]. Therefore, The combination of *IN* and *EX* features takes advantage of expiratory and inspiratory chest CT images.

In clinical practice, the primary difference between regular chest CT images and inspiratory and expiratory chest CT images is the dosage of contrast agents and the need for an additional scanning phase. Regular CT scans use a higher dose of contrast agent, which can potentially harm the patients and only capture images during inhalation. Therefore, inspiratory and expiratory chest CT scans are encouraged to reduce harm while maintaining diagnostic accuracy.

### 4.3 Lasso algorithm for AECOPD prediction feature selection

The Lasso algorithm considerably influenced our AECOPD prediction task. After the implementation of the Lasso algorithm, *IN*, *EX*, and their combination *IN* + *EX* features demonstrated a significant increase in performance. Specifically, the Lasso algorithm enhanced the performance of *IN* and *EX CNN/Radiomics* features. First, through dimensionality reduction of the dataset by feature selection, the Lasso algorithm effectively mitigates model complexity, thereby averting overfitting and enhancing generalization [69]. Second, Lasso serves to eliminate redundant or noisy features, mitigating model interference from irrelevant information and consequently improving overall performance [69]. Lastly, Lasso contributes to addressing multicollinearity, fostering model stability as an additional benefit [70]. Therefore, we can conclude that the Lasso algorithm helps predict AECOPD.

### 4.4 Meta-learning strategies for AECOPD prediction

The feasibility and effectiveness of meta-learning strategies show their potential application in the medical domain. The selected classic ML models have been widely used in COPD classification research [44]. Because few medical data lead to the classic ML models being difficult to train, so the meta-learning strategy is introduced [71]. The predictors based on the meta-learning strategy significantly outperformed ML models. Meanwhile, the AMGNN model performs better overall than the SNAIL model. Specifically, SNAIL based on *CNN/Radiomics* features slightly outperforms AMGNN. AMGNN based on the combination of *CNN* and *Radiomics* features outperforms SNAIL. This is mainly because the kernel of SNAIL is an attention mechanism (AM) that reallocates feature weights [71]. In contrast, the kernel of AMGNN is GNN, which effectively extracts relationships between patients on *CNN* and *Radiomics* features. The AM benefits from *CNN/Radiomics* features, but it fails in the combination of *CNN* and *Radiomics* features. This is because the combination of *CNN* and *Radiomics* features will affect the weight allocation between them. However, GNN extracts the relationships between different patients within the combination of *CNN* and *Radiomics* features, which is not affected by the variety of features. As a result, SNAIL's performance based on a combination of *CNN* and *Radiomics* features fails to improve compared to AMGNN.

### 4.5 Processing times and efficiency in AECOPD prediction

The runtime revealed notable variations in the computational efficiency across different techniques. The runtime of Med3D is much faster than PyRadiomics. This is because Med3D is a pre-trained feature extraction model compared to Pyradiomics. Compared to SNAIL and AMGNN predictors, ML predictors without CNN operation spend less time for training due to their simple structure. Meanwhile, compared to the SNAIL predictor, the AMGNN predictor spends less time on training and achieves better AECOPD prediction results. SNAIL consumes an amount of time due to the AM which requires the assignment of feature weights [71]. While SNAIL and AMGNN predictors necessitate longer training times compared to ML predictors, their superior performance suggests their potential as powerful tools in AECOPD prediction.

### 4.6 Exploring limitations and future directions in AECOPD research

While the AECOPD predictor based on *(EX + IN)_Lasso CNN* features has yielded promising results, there are also some limitations of our study. First, The AMGNN predictor needs to determine risk factors and node characteristics, which may make the prediction model inconvenient to use. Second, we do not conduct an in-depth analysis from a clinical perspective on the relationship between the features selected by the Lasso algorithm and the AECOPD prediction results. Third, this study was limited by inspiratory and expiratory chest CT images from a single center. Therefore, the proposed prediction model may need to be further validated on other real-world validations outside of this study's dataset. Besides, AECOPD results require a long-term follow-up track. Nevertheless, we have spent many years obtaining these chest CT images with AECOPD results. Last, it is necessary to focus on the CT imaging manifestations of AECOPD in future research directions, revealing the relationship between AECOPD and imaging.

## 5 Conclusions

The study proposes an AECOPD prediction model based on AMGNN and inspiratory and expiratory chest low-dose CT images. We have fully combined the features of inspiratory and expiratory chest CT images and adopted the currently advanced AMGNN predictor. The results indicate that AMGNN based on *(EX + IN)_Lasso CNN* features performs best, achieving an accuracy of 0.944, precision of 0.950, F1-score of 0.944, and area under the curve of 0.965. Therefore, it is concluded that our model may become an effective pre-clinical health management tool for AECOPD prediction.

## Data availability statement

The data supporting this study's findings are available from the corresponding author upon reasonable request.

## Ethics Statement

This study is approved by the Ethics Committee of the First Affiliated Hospital of Guangzhou Medical University (grant number:2017-22) and registered at the National Center for Biotechnology Information (NCT03240315" title="https://www.clinicaltrials.gov/study/ NCT03240315">https://www.clinicaltrials.gov/study/ NCT03240315, registration number: NCT03240315). All patients participating in the study were provided with written informed consent prior to the experiments. All patients consented to the use of CT images for publication.

## CRediT authorship contribution statement

**Shicong Wang:** Writing – original draft, Visualization, Validation, Software, Methodology, Formal analysis, Conceptualization. **Wei Li:** Writing – review & editing, Methodology, Formal analysis, Data curation. **Nanrong Zeng:** Software, Formal analysis. **Jiaxuan Xu:** Resources, Data curation. **Yingjian Yang:** Writing – review & editing, Conceptualization. **Xingguang Deng:** Software. **Ziran Chen:** Methodology. **Wenxin Duan:** Validation. **Yang Liu:** Software. **Yingwei Guo:** Writing – review & editing. **Rongchang Chen:** Resources, Data curation. **Yan Kang:** Writing – review & editing, Supervision, Project administration, Funding acquisition, Conceptualization.

## Declaration of competing interest

The authors declare that they have no known competing financial interests or personal relationships that could have appeared to influence the work reported in this paper.
